# Ultrashort-Chain and Short-Chain Per- and Polyfluoroalkyl Substances Are Universal Contaminants in Wines: A Comprehensive Survey of Red, White, Rosé, and Sparkling Samples

**DOI:** 10.3390/jox16050171

**Published:** 2026-09-11

**Authors:** Shun-Hsin Liang, Justin A. Steimling

**Affiliations:** Restek Corporation, 110 Benner Circle, Bellefonte, PA 16823, USA; justin.steimling@restek.com

**Keywords:** PFAS, ultrashort-chain PFAS, food safety, wine, trifluoroacetic acid (TFA)

## Abstract

Ultrashort-chain (USC) per- and polyfluoroalkyl substances (PFAS) are increasingly detected in environmental and drinking water systems, yet information regarding their occurrence in alcoholic beverages remains limited. The analysis of USC PFAS in wine is particularly challenging because of their high polarity and the complex composition of wine matrices. This study presents a streamlined analytical method for the simultaneous determination of 42 PFAS, including C1–C13 perfluoroalkyl carboxylic and sulfonic acids, in red, white, rosé, and sparkling wines. Chromatographic separation was achieved using a polar-embedded alkyl-phase liquid chromatography column, providing enhanced retention of USC PFAS while maintaining adequate performance for longer-chain analytes. A dilute-and-shoot sample preparation approach combined with 18 isotopically labeled internal standards enabled efficient analysis with minimal sample handling. Method verification using PFAS-spiked wine samples demonstrated satisfactory accuracy (recoveries within ±30% of nominal values) and precision (%RSD < 15%) across analyte-specific calibration ranges of 1–2500 ng/L. Application to commercial wines revealed the widespread occurrence of trifluoroacetic acid (TFA), trifluoromethanesulfonic acid (TFMS), perfluoropropionic acid (PFPrA), and perfluorobutanoic acid (PFBA) across all wine categories examined. These findings demonstrate the feasibility of comprehensive PFAS monitoring in wines and highlight the importance of including USC PFAS in exposure assessments of beverages.

## 1. Introduction

Per- and polyfluoroalkyl substances (PFAS) comprise a broad class of synthetic fluorinated chemicals widely used in industrial applications and consumer products because of their exceptional thermal stability and resistance to water, oil, and chemical degradation [1,2]. Their extensive historical and ongoing use has resulted in global environmental distribution and frequent detection in water, soil, air, biota, and food products [3]. Human exposure occurs through multiple pathways, including drinking water and diet, raising increasing concern regarding potential adverse health outcomes associated with selected PFAS compounds [4,5]. Although regulatory and monitoring efforts have historically focused on long-chain and legacy PFAS such as perfluorooctanoic acid (PFOA) and perfluorooctanesulfonic acid (PFOS), increasing attention is now being directed toward shorter-chain alternatives and ultrashort-chain (USC) PFAS.

USC PFAS generally refer to compounds containing fewer than four perfluorinated carbon atoms and include substances such as trifluoroacetic acid (TFA), perfluoropropionic acid (PFPrA), trifluoromethanesulfonic acid (TFMS), perfluoroethanesulfonic acid (PFEtS), and perfluoropropanesulfonic acid (PFPrS) [6,7]. These compounds are highly polar and highly mobile, and are often more difficult to remove during conventional treatment processes than longer-chain PFAS. Their occurrence has been increasingly documented in precipitation, rivers, groundwater, tap water, and drinking water supplies [8,9,10,11,12]. TFA, the most extensively studied USC PFAS, originates largely from atmospheric oxidation of hydrofluorocarbons (HFCs), hydrochlorofluorocarbons (HCFCs), and related fluorinated precursors, in addition to degradation of certain pesticides and pharmaceuticals [13,14,15,16]. PFPrA has also been associated with atmospheric transformation pathways and fluorinated precursor degradation [8,16], while TFMS may arise from industrial manufacturing processes or transformation of fluorinated materials [11,17]. PFEtS and PFPrS have been reported as constituents of aqueous film-forming foams (AFFF) and have been detected in groundwater contaminated by firefighting activities and military operations [18]. They may also arise as byproducts of electrochemical fluorination processes employed in the production of fluorinated surfactants and related compounds [19]. Because of their persistence and high water solubility, USC PFAS are increasingly recognized as important contributors to overall PFAS contamination and potential dietary exposure [6,12].

Despite growing awareness of USC PFAS, most analytical methods developed for dietary products monitoring continue to emphasize C4 and longer-chain compounds, potentially underestimating total PFAS occurrence. Recent studies have reported PFAS occurrence in bottled water, dairy products, and other beverages, although USC PFAS are frequently absent from target analyte lists [20]. Our group has recently expanded comprehensive PFAS analysis beyond drinking water to ready-to-drink teas, fruit juices, and liquid milk products through workflows specifically designed to include USC compounds [12,21,22]. These studies demonstrated that accurate determination of USC PFAS in beverage matrices requires contamination-controlled sample preparation and chromatographic conditions capable of retaining highly polar analytes while maintaining performance for longer-chain PFAS. A polar-embedded alkyl stationary phase has proven especially effective for this purpose, enabling simultaneous analysis of USC through long-chain PFAS under reversed-phase conditions within a practical run time.

Wine represents a particularly relevant yet underexplored beverage category for PFAS investigation. It is one of the most widely consumed alcoholic beverages worldwide, with global consumption exceeding 230 million hectoliters annually [23]. Wine is produced under diverse agricultural, fermentation, aging, and packaging conditions that may influence PFAS occurrence. Grapevines may be exposed to PFAS through irrigation water, soil amendments, atmospheric deposition, or pesticide use, while additional contamination may occur during vinification, filtration, storage, or bottling processes. Moreover, wine encompasses chemically diverse matrices. Red wines typically contain elevated levels of polyphenols, tannins, and pigments derived from skin fermentation; white wines generally possess lower phenolic content but higher acidity; rosé wines present intermediate composition; and sparkling wines introduce secondary fermentation effects, dissolved carbon dioxide, and variable sugar levels [24,25,26]. Collectively, these factors create analytical complexity and provide an ideal test set for evaluating method robustness across varied matrices.

Limited information is currently available regarding PFAS occurrence in wines. Published literatures have largely focused on TFA rather than broader PFAS target lists [27,28]. Accordingly, the aim of the present study was to develop and evaluate a robust and streamlined workflow for comprehensive PFAS determination across diverse wine matrices. A dilute-and-shoot sample preparation strategy was combined with LC-MS/MS analysis using a polar-embedded inert-coated alkyl-phase column to enable simultaneous quantification of USC through longer-chain PFAS in red, white, rosé, and sparkling wines. The analytical workflow was designed to quantify 42 PFAS representing multiple structural classes, including C2-C13 perfluoroalkyl carboxylic acids (PFCAs), C1–C13 perfluoroalkyl sulfonic acids (PFSAs), fluorotelomer carboxylic and sulfonic acids (FTCAs & FTSs), perfluorooctane sulfonamides (FOSAs), perfluorooctane sulfonamidoacetic acids (FOSAAs), and selected per- and polyfluoroether carboxylic and sulfonic acids. Method performance was rigorously evaluated for linearity, matrix effects, limits of detection and quantification, accuracy, and precision. The established workflow was then applied to commercial wine samples to characterize PFAS occurrence profiles and assess the importance of incorporating USC compounds into dietary exposure assessments. By extending comprehensive PFAS analysis to wines, this study addresses a pivotal data gap in complex liquid food matrices and contributes to a more complete understanding of PFAS contamination in the beverage supply.

## 2. Materials and Methods

### 2.1. Chemicals and Materials

Mobile phase components included LC/MS-grade acetonitrile (Fisher Scientific, Waltham, MA, USA) and reagent-grade formic acid and ammonium formate (Sigma-Aldrich, St. Louis, MO, USA). PFAS-grade acetonitrile and HPLC-grade methanol (Sigma-Aldrich, St. Louis, MO, USA) were utilized for standard dilution and sample preparation trials. In-house ultrapure deionized water (Restek Corporation, Bellefonte, PA, USA) was used for all aqueous solutions. Restek Corporation provided the following analytical components: the PFAS 28 calibration mixture, as well as the Ultra IBD Inert (100 × 2.1 mm, 3 µm) and Ultra IBD (150 × 2.1 mm, 3 µm) LC columns. Additional analytical standards, including an isotopically labeled mixture (MPFAC-24ES), two multi-component standards (PFAC-MXG and PFAC-MXI), and individual PFAS standards such as trifluoroacetic acid, perfluoropropanoic acid, sodium trifluoromethanesulfate, sodium perfluoroethanesulfonate, sodium perfluoropropanesulfonate, sodium perfluorounde-canesulfonate, sodium perfluorododecanesulfonate, sodium perfluorotridecanesulfonate, 3-perfluoropropyl propanoic acid, 3-perfluoropentyl propanoic acid, and 3-perfluoroheptyl propanoic acid, were sourced from Wellington Laboratories (Guelph, ON, Canada). Two isotope solutions, ^13^C_2_-trifluoroacetic acid and ^13^C_3_-perfluoropropanoic acid, were acquired from Cambridge Isotope Laboratories (Andover, MA, USA). Commercial wines were sourced from local retail outlets. Sample processing utilized 15-mL polypropylene centrifuge tubes from VWR International (Radnor, PA, USA). Standard solutions and final sample extracts were contained in 700-µL polypropylene vials with polyethylene caps from Waters Corporation (Milford, MA, USA).

### 2.2. Instrumentation and Chromatographic Method

Target analytes were analyzed using a Waters Acquity I-Class UPLC system interfaced with a Xevo TQ-S triple-quadrupole mass spectrometer (Waters Corporation, Milford, MA, USA). Detection was performed in negative electrospray ionization (ESI-) mode. Multiple reaction monitoring (MRM) transitions were optimized by evaluating precursor and fragment ion intensities to identify the most sensitive pathways. Quantitation was based on the primary (quantifier) transition, with a secondary (qualifier) transition utilized for identity confirmation where feasible. For specific ultrashort-chain and short-chain species, including TFA, PFPrA, PFBA, and PFPeA, a single product ion was monitored as no stable qualifier ions were observed. For HFPO-DA, the in-source fragmented ion [M–COOH]^−^ was selected as the precursor to maximize sensitivity under the optimized source conditions. Mass spectrometer operating parameters were maintained as follows: capillary voltage at 0.5 kV, desolvation temperature at 550 °C, and desolvation gas flow at 800 L/hr. The cone gas and collision gas flows were set to 150 L/hr and 0.20 mL/min, respectively, with a nebulizer pressure of 7.0 bar.

The 42 target analytes and 18 isotopically labeled internal standards were chromatographically separated using a Restek Ultra IBD Inert column (100 × 2.1 mm, 3 µm). To sequester system-related background contamination and ensure accurate quantitation, an Ultra IBD column (100 × 2.1 mm, 3.0 µm) was integrated into the flow path as a high-efficiency delay column. The aqueous mobile phase (A) consisted of ultrapure water containing 0.1% formic acid, while the organic mobile phase (B) consisted of acetonitrile/water (95:5, *v*/*v*) containing 2.5 mM ammonium formate and 0.1% formic acid. A gradient program was employed in which mobile phase B was initially set at 45%, ramped to 100% over 6 min, and maintained at that level through 10 min. The gradient was subsequently reset to the starting conditions, followed by column re-equilibration until the end of the 12-min run. The chromatographic system was operated at 30 °C with a flow rate of 0.40 mL/min and a 20-µL injection volume. Analyte retention times and corresponding optimized MRM transitions are provided in Table 1.

### 2.3. Standard and Sample Preparation

A mixed native standard solution containing all 42 target PFAS at 50 ng/mL was prepared in acetonitrile/water (1:1, *v*/*v*). Four analytes, PFHxS, PFOS, NMeFOSAA, and NEtFOSAA, were provided as technical mixtures containing both linear and branched isomers; accordingly, reported concentrations represent the combined response of all corresponding isomeric forms.

Calibration solutions covering the range of 1–2500 ng/L were prepared in polypropylene autosampler vials containing 500 µL of a 1:1 (*v*/*v*) ultrapure water/acetonitrile solution. Eighteen isotopically labeled PFAS were incorporated as quantitative internal standards (QIS; Table 1). To each calibration solution, 2.5 µL of a QIS mixture containing all labeled analytes at a concentration of 10 ng/mL was added.

For wine analysis, 0.5 mL aliquots of sample were transferred into 15 mL polypropylene centrifuge tubes. Samples were fortified with 5.0 µL of the QIS working solution and diluted with 0.5 mL acetonitrile. After vortex mixing for 30 s, the tubes were centrifuged at 4000 rpm to separate particulates and insoluble matrix components. The clarified supernatant was then transferred to polypropylene autosampler vials for LC-MS/MS analysis. This 1:1 dilution factor was accounted for in all final concentration calculations.

### 2.4. Control of Background Contamination and Blank Evaluation

Given the ubiquitous nature of PFAS and the potential for contamination from solvents, mobile phases, LC system components, and laboratory materials, stringent quality control procedures were incorporated throughout method development and sample analysis. To minimize interference from PFAS originating within the chromatographic system, a delay column was positioned between the pump and injector. As previously reported [21], a delay column packed with the same stationary phase (Ultra IBD) as the analytical column effectively retains system-derived PFAS, allowing contaminant signals, particularly those associated with long-chain compounds, to be chromatographically separated from analyte peaks.

To monitor potential contamination introduced during sample preparation, procedural blanks consisting of ultrapure water were prepared and analyzed alongside each sample batch using the same workflow. Instrument blanks were also periodically injected during analytical sequences to evaluate carryover and verify system cleanliness. Acceptance criteria required that PFAS responses observed in blank samples remain below the reporting limit or less than 30% of the signal measured at the lowest calibration standard.

In addition, laboratory consumables including pipette tips, centrifuge tubes, autosampler vials, and vial caps were pre-screened prior to use and selected based on their low PFAS background levels. Instrument performance was continuously monitored during analytical sequences through periodic injections of calibration standards and blank samples to confirm stable retention times, consistent detector response, and the absence of significant carryover.

## 3. Results

### 3.1. LC-MS/MS Method Development

The polar-embedded alkyl-phase Ultra IBD column has previously been employed successfully for the simultaneous analysis of USC PFAS and other PFAS compounds in water, milk, and beverage matrices [12,21,22]. Earlier work also showed that the inert-coated column configuration improved analytical sensitivity for many PFAS analytes. In the present study, this chromatographic platform was adapted for wine analysis, and its applicability was evaluated across a range of wine matrices characterized by distinct chemical compositions.

**Figure 1:**
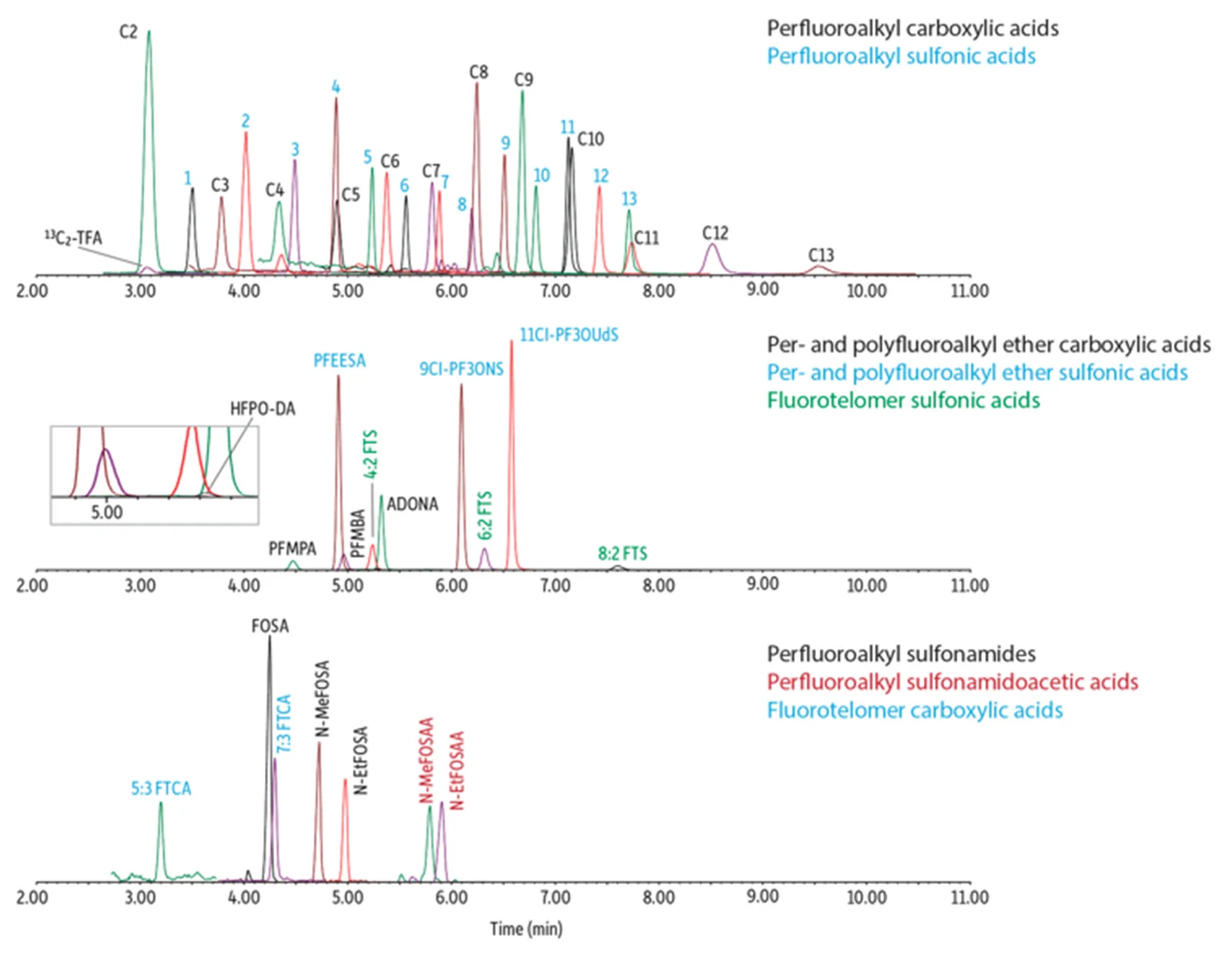
Chromatogram for the analysis of a 500 ng/L PFAS-spiked sparkling wine sample on the Ultra IBD Inert column (peak numbers correspond to the carbon chain lengths of the PFAS compounds).

Following preliminary assessment of chromatographic performance in red, white, rosé, and sparkling wine samples, LC conditions were further optimized from the earlier method to minimize wine-specific matrix interferences. Previous development studies with the Ultra IBD column showed that the combined use of ammonium formate and formic acid as mobile-phase additives was necessary to achieve satisfactory retention across C1–C13 PFAS while maintaining favorable peak shape and detection sensitivity. For wine analysis, both the concentration and distribution of these additives between the aqueous and organic mobile phases were adjusted to improve selectivity and separate matrix-derived interferences from all target analytes across the four wine classes. The resulting chromatographic performance is demonstrated in Figure 1, which displays a PFAS-spiked sparkling wine sample analyzed under the finalized, optimized conditions.

### 3.2. Sample Preparation Method Development

As highlighted in previous studies, minimizing sample handling is critical for reducing the risk of external contamination during comprehensive PFAS determination. Background contamination from TFA remains a particular concern because it is commonly encountered in solvents, reagents, and routine laboratory supplies. To reduce potential contamination, all consumables used during sample preparation, including pipette tips, autosampler vials, and centrifuge tubes, were pre-screened and selected based on their low PFAS background levels. Owing to the liquid nature of wine samples, the dilute-and-shoot strategy previously developed for liquid milk and beverage matrices [21,22] was adopted as the initial sample preparation approach for the present study.

In prior investigations, methanol was employed as the dilution solvent for water samples [12], while acetonitrile was found to be more suitable for the preparation of liquid milk and beverage matrices [21,22]. Therefore, both solvents were assessed in the present study to determine their influence on analyte recovery and chromatographic performance in wine samples. Analysis of PFAS-fortified wine samples revealed that long-chain PFAS produced generally higher and more consistent responses following a two-fold dilution with acetonitrile than with methanol. This observation indicates that acetonitrile more effectively maintained the solubility and stability of these hydrophobic compounds within the wine matrix. Based on these results, acetonitrile was selected as the dilution solvent for all subsequent analyses.

The robustness of this streamlined procedure was evaluated across a diverse suite of wine types. Following dilution, centrifugation was necessary to remove suspended particulates, particularly in red wine samples, in order to obtain a clear supernatant suitable for LC-MS/MS analysis. Finally, the injection volume was optimized to balance detection sensitivity with peak integrity. Larger injection volumes were associated with reduced retention and broader peaks for early-eluting analytes. A 20 µL injection volume provided the best compromise between sensitivity and chromatographic integrity and was therefore adopted for all wine samples.

### 3.3. Assessment of Matrix Effect, Extraction Recovery, and Extraction Efficiency

To evaluate matrix effects, one representative sample from each wine category was selected to capture a broad range of matrix characteristics, including differences in acidity, residual sugar, pigment content, polyphenols, and tannins that could influence electrospray ionization behavior. Blank sample extracts were prepared using the finalized dilute-and-shoot procedure and subsequently fortified to 200 ng/L (post-spiked extracts) prior to LC-MS/MS analysis. Matrix effects were determined by comparing analyte peak areas in the post-spiked extracts with those obtained from a standard solution prepared at the same concentration. Because all wine samples contained substantial endogenous levels of TFA, direct evaluation of native TFA was not feasible. Therefore, ^13^C_2_-TFA was used as a surrogate to assess matrix effects for TFA, as this isotopically labeled analogue was confirmed to exhibit MS behavior comparable to the native compound.

As summarized in Supplementary Materials Table S1, perfluoroalkyl carboxylic acids with carbon chain lengths from C2 to C10 showed notable signal suppression across all wine types. Comparable suppression trends were also observed for fluorotelomer carboxylic acids, perfluorooctane sulfonamides, sulfonamidoacetic acids, and selected perfluoroether carboxylic acids. In contrast, perfluoroalkyl sulfonic acids were generally less affected by matrix-induced suppression. Perfluoroethanesulfonic acid (PFEtS), the C2 sulfonic acid, was the only analyte that exhibited signal enhancement in white, rosé, and sparkling wines. These findings demonstrate that wine matrices can impose substantial and analyte-dependent ionization effects, highlighting the importance of implementing isotopically labeled quantitative internal standards (QIS) for accurate compensation of matrix-related bias.

For recovery studies, representative wine samples were fortified at 400 ng/L prior to sample preparation and then processed using the finalized extraction procedure (pre-spiked extracts). Extraction recovery was estimated by comparing analyte peak areas in the pre-spiked extracts with those of standard solutions at the equivalent final concentration (200 ng/L). Extraction efficiency was determined by comparing analyte responses in pre-spiked extracts with those measured in corresponding post-spiked extracts. Overall, extraction efficiency for the majority of analytes was consistent among the four wine categories, indicating that the dilute-and-shoot workflow provided reliable and reproducible analyte transfer across diverse wine matrices.

### 3.4. Evaluation of Method Accuracy and Precision

Method performance was evaluated in terms of accuracy and precision using four representative wine matrices spiked with native PFAS at concentrations of 2, 4, 10, 20, 100, and 500 ng/L. Recovery of TFA was assessed using isotopically labeled ^13^C_2_-TFA as a surrogate standard, while a suite of 18 isotopically labeled PFAS compounds was added to each fortified sample as QIS. Validation experiments were conducted across three independent analytical runs performed on separate days, with three replicate sample preparations at each spiking level in each run, yielding a total of nine replicates per concentration level. Since TFMS, PFPrA, and PFBA were present in all wine samples as incurred residues, their background concentrations were measured in corresponding unfortified samples and deducted from the fortified sample results before recovery calculations were performed.

To establish a broadly applicable quantification strategy for diverse wine matrices, significant effort was invested in optimizing the assignment of isotopically labeled QIS to their corresponding native analytes. The pronounced variability in matrix effects observed among the four representative wine types made it difficult to identify a single set of QIS-analyte pairings that would perform adequately across all matrices. After comprehensive evaluation, the final assignments presented in Table 1 were selected and applied uniformly to all wine samples. This approach effectively compensated for matrix-dependent signal variations and enabled reliable, accurate quantification across a wide range of wine matrices.

Calibration curves were constructed using quadratic regression with 1/x weighting. Excellent linearity was achieved for all analytes, with correlation coefficients (r^2^) exceeding 0.995 and calibration point deviations remaining within ±30%. For most analytes, the calibration range spanned 1–1000 ng/L or 2–1000 ng/L, while ranges of 10–1000 ng/L for HFPO-DA, 10–2500 ng/L for TFA, and 4–1000 ng/L for several other compounds were established as appropriate (Supplementary Materials Table S2). The limit of quantification (LOQ) was defined as the lowest fortification level that satisfied predefined accuracy and precision acceptance criteria. The limit of detection (LOD) was estimated from fortified wine samples based on a signal-to-noise ratio of 3 (Supplementary Materials Table S2).

Average recoveries and relative standard deviation (%RSD) values are summarized in Supplementary Materials Table S3. Excluding PFEtS, recoveries ranged from 84.9–113% in red wine, 81.7–116% in white wine, 82.5–114% in rosé wine, and 87.4–119% in sparkling wine. Because PFEtS exhibited signal enhancement and no suitable isotopically labeled analogue was available for optimal correction, its apparent recoveries were higher, reaching approximately 150% in some matrices. Nevertheless, satisfactory method precision was achieved, with %RSD values below 15% for all wine categories.

Overall, most perfluoroalkyl sulfonic acids, fluorotelomer sulfonic acids, and perfluoroether sulfonic acids were reliably quantified at an LOQ of 2 ng/L across all wine matrices evaluated. Most of the remaining target analytes achieved LOQs of 4 ng/L. Higher LOQs (10–20 ng/L) were required for selected compounds, including TFA (quantified using ^13^C_2_-TFA), HFPO-DA, fluorotelomer carboxylic acids, perfluorooctane sulfonamides, and sulfonamidoacetic acids, primarily because of lower mass spectrometric sensitivity and/or greater matrix interference, depending on the wine type. Endogenous concentrations of TFMS, PFPrA, and PFBA were detected in all wine samples, limiting verification of lower quantification levels; therefore, their practical LOQs were established at 10 ng/L following background correction. Despite these limitations, the majority of the 42 target PFAS were accurately and precisely quantified at concentrations of 2–4 ng/L, demonstrating that the developed dilute-and-shoot workflow provides a robust, sensitive, and efficient approach for comprehensive PFAS analysis in diverse wine matrices.

### 3.5. PFAS Contamination Profiles in an Expanded Selection of Commercial Wines

The established analytical workflow was applied to the determination of PFAS in 25 commercially available wines obtained from local retail sources. The sample set comprised 10 red wines and 5 samples each of white, rosé, and sparkling wines, representing products from multiple global wine-producing regions, including Australia, France, Italy, New Zealand, and the United States. All samples were analyzed in duplicate to ensure data reliability.

A highly consistent contamination pattern was observed across all samples. Four PFAS compounds, TFMS, TFA, PFPrA, and PFBA, were detected in every wine analyzed, while no additional PFAS from the target list were identified (Supplementary Materials Table S4). Because TFA, PFPrA, and PFBA each produce only a single MS/MS transition, additional confirmation was required to verify that the detected peaks were true analyte signals rather than matrix-related interferences. To confirm their identity, wine samples were analyzed using an alternative LC method employing a substantially different mobile phase composition and gradient program (Supplementary Materials Table S5), which resulted in markedly increased retention times for these three analytes. Under these modified chromatographic conditions, TFA, PFPrA, and PFBA remained detectable in the wine samples, and their peaks still co-eluted with the corresponding isotopically labeled internal standards (Supplementary Materials Figure S1). These results confirm that the detected signals originated from the target analytes rather than chromatographic artifacts or matrix noise.

TFA was present at substantially higher concentrations than the other analytes, ranging from 20,777 to 406,350 ng/L. Preliminary analyses indicated that TFA concentrations exceeded the upper limit of the calibration range; therefore, samples were diluted 50-fold with ultrapure water prior to application of the dilute-and-shoot procedure to ensure accurate quantification. The remaining three compounds were detected at relatively lower concentrations in the ng/L range. TFMS was observed between 6 and 70 ng/L, PFPrA ranged from 29 to 334 ng/L, and PFBA ranged from 14 to 400 ng/L. Despite the diversity of wine types and geographic origins, no clear trends in PFAS concentrations or distribution patterns were observed among red, white, rosé, and sparkling wines or across different production regions.

## 4. Discussion

The consistent detection of TFMS, TFA, PFPrA, and PFBA, across all wine samples suggests a common set of environmental and physicochemical drivers governing PFAS occurrence in this matrix. Notably, all four compounds belong to ultrashort- and short-chain PFAS, which are characterized by high water solubility, low sorption to organic matter, and enhanced environmental mobility. The absence of longer-chain PFAS further emphasizes the importance of including USC compounds in analytical workflows, as conventional PFAS methods may overlook the dominant contributors to total PFAS burden in such matrices.

One key factor contributing to their presence in wines is plant uptake. Short-chain PFAS are known to be more readily taken up and translocated within plants compared to longer-chain analogues. Studies have demonstrated that the short-chain compound PFBA exhibits significantly higher bioconcentration factors in edible plant tissues such as lettuce and tomato, with efficient root-to-shoot transport [29,30]. This behavior is attributed to their lower hydrophobicity and reduced binding affinity to soil organic matter, enabling greater bioavailability in soil pore water. In the context of viticulture, grapevines may similarly accumulate these highly mobile PFAS from irrigation water, soil, and atmospheric deposition, leading to their incorporation into grapes and subsequently into wine.

Atmospheric deposition represents another important pathway, particularly for TFA and PFPrA. These compounds are well-documented end products of atmospheric oxidation of hydrofluorocarbons (HFCs) and related fluorinated precursors [13,14,15,16]. TFA, in particular, has been widely reported in precipitation and surface waters worldwide, often as the dominant PFAS species [8]. PFPrA has also been identified as a major contributor to PFAS in rainwater, accounting for a substantial fraction of total PFAS flux via wet deposition. This widespread atmospheric presence provides a continuous input source to agricultural systems, including vineyards. It should also be recognized that wine packaging materials may represent an additional source of PFAS contamination. The potential contribution of bottle glass, closures, cap liners, sealing materials, and other packaging components cannot be completely excluded. Therefore, while atmospheric deposition is likely an important source of TFA in wines, additional studies specifically evaluating PFAS migration from packaging materials would be valuable, particularly for samples exhibiting elevated TFA concentrations. In contrast, longer-chain PFAS are more strongly retained in soils due to hydrophobic interactions and sorption to organic carbon, limiting their uptake into plants and transfer into fruit matrices [31]. This preferential retention reduces their bioavailability and helps explain their absence in the analyzed wine samples. Additionally, longer-chain PFAS are more likely to associate with particulate matter and may be removed during wine production processes such as clarification, filtration, and aging. The multistep nature of wine production may therefore further decrease the concentrations of longer-chain PFAS in the final product, providing a plausible explanation for why ultrashort-chain and short-chain PFAS were the predominant compounds detected in the wines analyzed in this study.

One limitation of the present study is the unequal distribution of samples among the wine categories evaluated. Although the survey included red, white, rosé, and sparkling wines, the number of samples within each category varied, which may have reduced the statistical power of between-category comparisons. Consequently, differences in PFAS concentrations among wine types should be interpreted with caution. Future studies incorporating larger and more balanced sample sets across wine categories, geographic regions, and production characteristics would help strengthen statistical comparisons and further elucidate factors influencing PFAS occurrence in wines.

The TFA concentrations observed in this study (20,777 to 406,350 ng/L) were substantially higher than those of the other PFAS detected. Assuming a daily wine consumption of 150 mL, the estimated daily intake of TFA from wine would range from approximately 3120 to 60,950 ng/day based on the concentration range observed in the surveyed samples. Although wine is unlikely to represent the sole source of dietary TFA exposure, these findings demonstrate that wine may contribute measurably to overall TFA intake. Notably, the TFA concentrations measured in these wine samples are approximately 40- to 800-fold higher than the 500 ng/L concentration currently being discussed as a potential future regulatory limit for TFA in drinking water in the European Union. While direct comparisons between wine and drinking water should be interpreted cautiously because of differences in consumption patterns and regulatory frameworks, the elevated TFA levels observed in wine highlight the importance of evaluating food and beverage contributions to overall human TFA exposure. Given the increasing regulatory and scientific attention directed toward TFA, further research examining its occurrence across a broader range of food and beverage products would be valuable for refining dietary exposure assessments and better understanding of potential public health implications.

Collectively, these factors, including environmental prevalence, atmospheric inputs, and plant uptake behavior, provide a coherent explanation for the universal presence of TFA, PFPrA, PFBA, and TFMS in wines. These findings are consistent with growing evidence that ultrashort- and short-chain PFAS represent dominant contributors to PFAS burdens in aqueous food systems and highlight the importance of including these compounds in future exposure and risk assessments.

## 5. Conclusions

This study developed a simple and robust workflow for the comprehensive determination of ultrashort- to long-chain PFAS in diverse wine matrices. The method enabled reliable quantification of 42 PFAS while effectively addressing matrix complexity. Application to 25 commercial wines revealed a consistent contamination profile, with TFMS, TFA, PFPrA, and PFBA detected in all samples. TFA was present at substantially higher concentrations, while no longer-chain PFAS were observed. These findings demonstrate that ultrashort- and short-chain PFAS dominate contamination in wines and must be included in analytical methods to ensure accurate exposure assessment. The proposed workflow provides a practical tool for routine PFAS monitoring in complex beverage matrices.

## Figures and Tables

**Table 1:** MS ion transitions, parameters, and chromatographic retention time of analytes.

Compounds	Retention	Precursor Ion	Product Ions *^a^*	Cone (V)	Collision (V)	Quantification
	Time (min)					Internal Standard
*Target Analytes*						
** Perfluoroalkyl carboxylic acids**						
Trifluoroacetic acid (TFA)	3.15	113.03 [M-H]^−^	69.01	10	10	d3-N-MeFOSAA
Perfluoropropanoic acid (PFPrA)	3.84	162.97 [M-H]^−^	119.02	10	8	^13^C_3_-PFPrA
Perfluorobutanoic acid (PFBA)	4.59	213.03 [M-H]^−^	168.98	14	8	^13^C_4_-PFBA
Perfluoropentanoic acid (PFPeA)	4.95	262.97 [M-H]^−^	218.97	2	6	^13^C_5_-PFPeA
Perfluorohexanoic acid (PFHxA)	5.43	313.10 [M-H]^−^	268.97/118.99	2	8/20	^13^C_5_-PFHxA
Perfluoroheptanoic acid (PFHpA)	5.86	363.16 [M-H]^−^	319.09/169.06	8	10/18	^13^C_4_-PFHpA
Perfluorooctanoic acid (PFOA)	6.29	413.10 [M-H]^−^	368.96/168.90	2	10/16	^13^C_8_-PFOA
Perfluorononanoic acid (PFNA)	6.73	463.10 [M-H]^−^	419.01/219.02	4	10/16	^13^C_9_-PFNA
Perfluorodecanoic acid (PFDA)	7.20	513.17 [M-H]^−^	469.16/219.06	4	12/16	^13^C_6_-PFDA
Perfluoroundecanoic acid (PFUnA)	7.76	563.23 [M-H]^−^	519.24/269.07	6	12/18	^13^C_2_-PFDoA
Perfluorododecanoic acid (PFDoA)	8.55	613.23 [M-H]^−^	569.19/169.06	8	12/26	^13^C_2_-PFDoA
Perfluorotridecanoic acid (PFTrDA)	9.55	663.23 [M-H]^−^	619.21/169.06	8	14/28	^13^C_2_-PFDoA
**Perfluoroalkyl sulfonic acids**						
Trifluoromethanesulfonic acid (TFMS)	3.57	148.97 [M-H]^−^	79.93/98.92	62	13/18	^13^C_3_-PFBS
Perfluoroethanesulfonic acid (PFEtS)	4.08	198.90 [M-H]^−^	79.92/98.91	38	22/22	^13^C_3_-PFBS
Perfluoropropanesulfonic acid (PFPrS)	4.55	248.97 [M-H]^−^	79.92/98.91	2	24/24	^13^C_3_-PFBS
Perfluorobutanesulfonic acid (PFBS)	4.94	298.97 [M-H]^−^	79.97/98.89	2	26/26	^13^C_3_-PFBS
Perfluoropentanesulfonic acid (PFPeS)	5.29	349.10 [M-H]^−^	79.98/98.98	6	32/30	^13^C_3_-PFHxS
Perfluorohexanesulfonic acid (PFHxS)	5.62	398.90 [M-H]^−^	79.97/98.89	56	32/34	^13^C_3_-PFHxS
Perfluoroheptanesulfonic acid (PFHpS)	5.93	449.17 [M-H]^−^	79.98/98.97	4	42/38	^13^C_8_-PFOS
Perfluorooctanesulfonic acid (PFOS)	6.24	499.03 [M-H]^−^	79.92/98.90	8	40/40	^13^C_8_-PFOS
Perfluorononanesulfonic acid (PFNS)	6.55	549.10 [M-H]^−^	79.92/98.83	12	42/40	^13^C_3_-PFHxS
Perfluorodecanesulfonic acid (PFDS)	6.85	599.17 [M-H]^−^	79.98/98.83	8	44/46	^13^C_3_-PFHxS
Perfluoroundecanesulfonic acid (PFUdS)	7.16	648.73 [M-H]^−^	79.94/98.94	38	50/44	^13^C_3_-PFHxS
Perfluorododecanesulfonic acid (PFDoS)	7.46	698.77 [M-H]^−^	79.95/98.94	10	60/44	^13^C_3_-PFHxS
Perfluorotridecanesulfonic acid (PFTrDS)	7.75	748.73 [M-H]^−^	79.94/98.94	8	76/52	^13^C_3_-PFHxS
**Fluorotelomer sulfonic acids**						
1H,1H,2H,2H-Perfluorohexane sulfonic acid (4:2 FTS)	5.27	327.10 [M-H]^−^	307.08/80.83	50	18/24	^13^C_2_-4:2FTS
1H,1H,2H,2H-Perfluorooctane sulfonic acid (6:2 FTS)	6.35	427.17 [M-H]^−^	407.18/80.71	2	22/32	^13^C_2_-6:2FTS
1H,1H,2H,2H-Perfluorodecane sulfonic acid (8:2 FTS)	7.64	527.17 [M-H]^−^	507.16/80.83	66	26/32	^13^C_2_-8:2FTS
**Fluorotelomer carboxylic acids**						
3-perfluoropentyl propanoic acid (5:3 FTCA)	3.21	340.93 [M-H]^−^	216.96/236.93	2	24/14	^13^C_5_-PFHxA
3-perfluoroheptyl propanoic acid (7:3 FTCA)	4.29	440.90 [M-H]^−^	336.88/316.91	20	12/22	^13^C_4_-PFHpA
**Perfluoroalkyl sulfonamides**						
Perfluorooctanesulfonamide (FOSA)	4.24	498.17 [M-H]^−^	77.97/477.76	8	28/26	^13^C_8_-FOSA
N-methyl perfluorooctanesulfonamide (NMeFOSA)	4.71	511.77 [M-H]^−^	168.95/218.91	2	26/24	^13^C_5_-PFPeA
N-ethyl perfluorooctanesulfonamide (NEtFOSA)	4.96	525.83 [M-H]^−^	168.96/218.92	10	26/24	^13^C_5_-PFPeA
**Perfluoroalkyl sulfonamidoacetic acids**						
N-methyl perfluorooctanesulfonamidoacetic acid (NMeFOSAA)	5.76	570.20 [M-H]^−^	419.17/483.16	46	20/14	d3-N-MeFOSAA
N-ethyl perfluorooctanesulfonamidoacetic acid (NEtFOSAA)	5.87	584.20 [M-H]^−^	419.18/483.11	6	20/16	d5-N-EtFOSAA
**Per- and Polyfluoroether carboxylic acids**						
Perfluoro-3-methoxypropanoic acid (PFMPA)	4.49	228.93 [M-H]^−^	84.97/198.94	10	10/14	^13^C_4_-PFBA
Perfluoro-4-methoxybutanoic acid (PFMBA)	4.99	278.87 [M-H]^−^	84.96/234.93	8	10/6	^13^C_5_-PFPeA
Hexafluoropropylene oxide dimer acid (HFPO-DA)	5.31	285.03 [M-COOH]^−^	169.02/185.02	2	6/16	^13^C_3_-PFBS
4,8-Dioxa-3H-perfluorononanoic acid (ADONA)	5.36	376.90 [M-H]^−^	250.93/84.97	22	12/26	^13^C_5_-PFHxA
**Per- and Polyfluoroether sulfonic acids**						
Perfluoro(2-ethoxyethane)sulfonic acid (PFEESA)	4.94	314.83 [M-H]^−^	134.94/83.01	4	22/16	^13^C_3_-PFBS
9-Chlorohexadecafluoro-3-oxanonane-1-sulfonic acid (9Cl-PF3ONS)	6.13	530.78 [M-H]^−^	350.85/82.96	12	26/24	^13^C_8_-PFOS
11-Chloroeicosafluoro-3-oxaundecane-1-sulfonic acid (11Cl-PF3OUdS)	6.61	630.78 [M-H]^−^	450.80/82.95	8	26/32	^13^C_3_-PFBS
***Surrogate***						
^13^C_2_-TFA	3.33	114.90 [M-H]^−^	69.95	14	8	d3-N-MeFOSAA
***Quantification Internal Standards***						
^13^C_3_-PFPrA	4.05	165.97 [M-H]^−^	120.96	10	11	
^13^C_4_-PFBA	4.59	217.03 [M-H]^−^	171.98	2	8	
^13^C_5_-PFPeA	5.10	267.97 [M-H]^−^	222.99	2	6	
^13^C_5_-PFHxA	5.54	318.03 [M-H]^−^	272.93	2	7	
^13^C_4_-PFHpA	5.95	366.90 [M-H]^−^	321.93	2	10	
^13^C_8_-PFOA	6.37	420.97 [M-H]^−^	375.94	2	10	
^13^C_9_PFNA	6.80	471.97 [M-H]^−^	426.87	4	12	
^13^C_6_-PFDA	7.29	518.90 [M-H]^−^	473.87	4	13	
^13^C_2_-PFDoA	8.73	714.78 [M-H]^−^	669.80	8	14	
^13^C_3_-PFBS	5.03	301.97 [M-H]^−^	79.97	2	28	
^13^C_3_-PFHxS	5.68	401.90 [M-H]^−^	79.97	2	36	
^13^C_8_-PFOS	6.30	506.84 [M-H]^−^	79.97	4	42	
^13^C_8_-FOSA	4.23	505.91 [M-H]^−^	77.95	4	32	
^13^C_2_-4:2FTS	5.41	328.97 [M-H]^−^	308.96	2	18	
^13^C_2_-6:2FTS	6.46	428.97 [M-H]^−^	408.90	2	24	
^13^C_2_-8:2FTS	7.79	528.90 [M-H]^−^	508.90	2	24	
d3-N-MeFOSAA	5.82	572.90 [M-H]^−^	418.91	50	18	
d5-N-EtFOSAA	5.93	588.97 [M-H]^−^	418.86	48	20	

*^a^* Quantifier Ion/Qualifier Ion.

## Data Availability

The original contributions presented in this study are included in the article/Supplementary Materials. Further inquiries can be directed to the corresponding author.

## Supplementary Materials

The following supporting information can be downloaded at: https://www.mdpi.com/article/10.3390/jox16050171/s1, Table S1: Matrix effect, extraction recovery, and extraction efficiency of varying wine types; Table S2: Linearity and limit of detection; Table S3: Accuracy and precision for the analysis of fortified wine samples; Table S4: Measurement of PFAS in commercial wine samples; Table S5: Alternative LC method for confirmatory analysis of TFA, PFPrA, and PFBA in wine; Figure S1: Confirmatory chromatographic comparison of original and alternative LC methods for TFA, PFPrA, and PFBA in a red wine sample.
