# Supplementary material for: Ultrashort-Chain and Short-Chain Per- and Polyfluoroalkyl Substances Are Universal Contaminants in Wines: A Comprehensive Survey of Red, White, Rosé, and Sparkling Samples
Source: J Xenobiot. 2026 Sep 11;16(5):171. doi: 10.3390/jox16050171 (PMC13608146; doi:10.3390/jox16050171)

# Supplementary Materials: Ultrashort-Chain and Short-Chain Per- and Polyfluoroalkyl Substances Are Universal Contaminants in Wines: A Comprehensive Survey of Red, White, Rosé, and Sparkling Samples

**Shun-Hsin Liang and Justin A. Steimling**

**Table S1.** Matrix Effect, extraction recovery, and extraction efficiency of varying wine types

| | Red Wine | | | White Wine | | | Rosé Wine | | | Sparkling Wine | | |
|---|---|---|---|---|---|---|---|---|---|---|---|---|
| Analytes | Matrix Effect[1] | Extraction Recovery[2] | Extraction Efficiency[3] | Matrix Effect[1] | Extraction Recovery[2] | Extraction Efficiency[3] | Matrix Effect[1] | Extraction Recovery[2] | Extraction Efficiency[3] | Matrix Effect[1] | Extraction Recovery[2] | Extraction Efficiency[3] |
| $^{13}C_2$-TFA | 33.2 | 30.5 | 92.0 | 41.2 | 38.7 | 94.0 | 35.4 | 31.7 | 89.5 | 48.5 | 41.2 | 86.5 |
| PFPrA | 66.3 | 60.0 | 90.4 | 93.8 | 86.5 | 92.2 | 87.3 | 85.4 | 97.9 | 76.3 | 69.0 | 90.4 |
| PFBA | 59.1 | 52.1 | 88.0 | 80.6 | 73.6 | 91.3 | 74.6 | 73.2 | 98.2 | 72.3 | 65.4 | 90.4 |
| PFPeA | 49.5 | 46.3 | 93.6 | 74.0 | 69.6 | 94.1 | 51.3 | 48.4 | 94.3 | 67.6 | 58.4 | 86.3 |
| PFHxA | 48.1 | 44.6 | 92.8 | 61.4 | 56.9 | 92.7 | 50.7 | 48.5 | 95.6 | 58.5 | 55.5 | 94.8 |
| PFHpA | 47.4 | 42.6 | 89.9 | 53.3 | 46.2 | 86.7 | 42.5 | 41.2 | 97.0 | 61.7 | 55.0 | 89.1 |
| PFOA | 44.4 | 37.2 | 83.8 | 54.8 | 47.7 | 87.1 | 50.4 | 45.2 | 89.8 | 65.2 | 54.1 | 83.0 |
| PFNA | 52.3 | 45.5 | 86.9 | 70.0 | 60.5 | 86.4 | 60.1 | 55.4 | 92.2 | 80.7 | 66.8 | 82.8 |
| PFDA | 67.7 | 60.9 | 90.0 | 82.1 | 73.8 | 89.9 | 72.2 | 69.5 | 96.2 | 72.3 | 61.5 | 85.1 |
| PFUnA | 83.4 | 73.9 | 88.6 | 97.1 | 84.6 | 87.1 | 87.8 | 81.8 | 93.2 | 97.8 | 86.5 | 88.5 |
| PFDoA | 82.2 | 76.0 | 92.5 | 98.4 | 87.3 | 88.7 | 90.4 | 83.7 | 92.5 | 98.2 | 85.7 | 87.3 |
| PFTrDA | 83.5 | 75.6 | 90.5 | 98.1 | 86.5 | 88.2 | 89.4 | 85.4 | 95.5 | 101 | 87.3 | 86.8 |
| TFMS | 76.9 | 67.8 | 88.2 | 87.5 | 82.0 | 93.7 | 81.0 | 77.3 | 95.4 | 87.8 | 77.1 | 87.7 |
| PFEtS | 98.6 | 92.7 | 94.1 | 128 | 118 | 92.2 | 124 | 119 | 96.5 | 120 | 109 | 91.0 |
| PFPrS | 67.0 | 64.3 | 95.9 | 66.1 | 57.6 | 87.1 | 63.3 | 60.2 | 95.1 | 84.2 | 74.4 | 88.3 |
| PFBS | 76.6 | 70.0 | 91.4 | 87.2 | 81.5 | 93.4 | 81.3 | 79.7 | 98.1 | 92.4 | 78.5 | 84.9 |
| PFPeS | 67.2 | 61.4 | 91.3 | 84.2 | 76.0 | 90.3 | 71.5 | 69.5 | 97.2 | 85.1 | 75.4 | 88.7 |
| PFHxS | 68.8 | 65.7 | 95.5 | 78.7 | 72.8 | 92.5 | 76.6 | 76.0 | 99.2 | 92.0 | 78.7 | 85.5 |
| PFHpS | 85.3 | 80.1 | 93.9 | 89.7 | 83.9 | 93.5 | 78.1 | 77.0 | 98.6 | 86.0 | 79.4 | 92.3 |
| PFOS | 79.6 | 73.7 | 92.7 | 93.7 | 85.8 | 91.6 | 82.6 | 75.2 | 91.1 | 89.3 | 76.2 | 85.3 |
| PFNS | 73.0 | 66.5 | 91.1 | 88.2 | 76.6 | 86.8 | 79.9 | 75.0 | 93.8 | 89.1 | 78.4 | 88.0 |
| PFDS | 85.9 | 81.5 | 94.9 | 93.3 | 85.1 | 91.3 | 85.1 | 84.1 | 98.8 | 94.5 | 85.1 | 90.0 |
| PFUdS | 82.2 | 74.4 | 90.5 | 91.4 | 82.3 | 90.1 | 86.7 | 83.1 | 95.8 | 94.3 | 83.9 | 88.9 |
| PFDoS | 81.7 | 75.2 | 92.1 | 92.8 | 81.1 | 87.4 | 88.7 | 84.4 | 95.2 | 98.2 | 84.2 | 85.8 |
| PFTrDS | 80.6 | 73.8 | 91.5 | 94.4 | 81.6 | 86.4 | 86.7 | 81.7 | 94.3 | 97.8 | 84.9 | 86.8 |
| 4:2 FTS | 54.5 | 50.0 | 91.6 | 59.9 | 51.5 | 86.0 | 25.9 | 25.1 | 96.8 | 56.0 | 48.2 | 86.1 |

| | | | | | | | | | | | | |
|---|---|---|---|---|---|---|---|---|---|---|---|---|
| 6:2 FTS | 70.4 | 66.5 | 94.5 | 55.5 | 46.9 | 84.5 | 50.5 | 49.9 | 98.7 | 70.8 | 59.6 | 84.2 |
| 8:2 FTS | 89.5 | 81.4 | 91.0 | 99.2 | 88.6 | 89.4 | 87.9 | 86.3 | 98.1 | 107 | 92.1 | 86.1 |
| 5:3 FTCA | 43.0 | 37.5 | 87.2 | 52.8 | 48.2 | 91.1 | 40.8 | 36.4 | 89.2 | 52.1 | 44.3 | 85.0 |
| 7:3 FTCA | 39.1 | 33.9 | 86.8 | 70.8 | 60.6 | 85.5 | 54.7 | 48.4 | 88.6 | 65.8 | 50.2 | 76.3 |
| FOSA | 45.2 | 42.9 | 94.8 | 67.9 | 61.7 | 90.9 | 56.0 | 53.0 | 94.6 | 58.5 | 49.9 | 85.3 |
| NMeFOSA | 39.5 | 34.2 | 86.6 | 75.5 | 72.2 | 95.6 | 72.0 | 65.3 | 90.7 | 78.1 | 64.3 | 82.3 |
| NEtFOSA | 43.7 | 39.3 | 89.9 | 78.9 | 68.7 | 87.0 | 58.2 | 53.3 | 91.7 | 67.1 | 58.1 | 86.6 |
| NMeFOSAA | 38.8 | 30.6 | 78.9 | 35.5 | 34.3 | 96.5 | 34.4 | 31.8 | 92.3 | 52.8 | 45.0 | 85.2 |
| NEtFOSAA | 43.7 | 39.3 | 89.9 | 38.6 | 41.2 | 106.8 | 33.0 | 29.2 | 88.5 | 55.2 | 45.1 | 81.8 |
| ADONA | 44.5 | 40.2 | 90.4 | 61.4 | 54.9 | 89.3 | 45.2 | 44.7 | 98.8 | 60.2 | 51.7 | 85.9 |
| HFPO-DA | 80.6 | 75.7 | 93.9 | 90.5 | 80.6 | 89.0 | 86.8 | 66.5 | 76.5 | 82.8 | 85.3 | 103 |
| NFDHA | 87.9 | 90.6 | 103 | 118 | 84.4 | 71.3 | 61.3 | 54.4 | 88.8 | 64.2 | 42.9 | 66.8 |
| PFMPA | 47.2 | 43.9 | 93.0 | 69.3 | 61.3 | 88.4 | 54.8 | 53.0 | 96.8 | 62.9 | 54.8 | 87.1 |
| PFMBA | 52.8 | 46.6 | 88.3 | 69.1 | 64.2 | 92.8 | 51.9 | 51.8 | 99.9 | 59.2 | 54.7 | 92.3 |
| PFEESA | 69.9 | 64.0 | 91.6 | 86.1 | 78.0 | 90.5 | 77.6 | 74.8 | 96.3 | 87.0 | 78.5 | 90.3 |
| 9Cl-PF3ONS | 67.9 | 60.6 | 89.3 | 81.2 | 75.1 | 92.5 | 68.0 | 65.8 | 96.8 | 75.8 | 65.8 | 86.8 |
| 11Cl-PF3OUdS | 76.7 | 69.7 | 90.8 | 88.7 | 79.0 | 89.1 | 82.9 | 79.7 | 96.1 | 93.3 | 81.9 | 87.8 |

[1] (peak area of post-spiked extract)/(peak area of standard solution) x 100

[2] (peak area of pre-spiked extract)/(peak area of standard solution) x 100

[3] (peak area of pre-spiked extract)/(peak area of post-spiked extract) x 100

**Table S2**. Linearity and limit of detection

| Analytes | Linearity Range[1] (ng/L) | LOD (ng/L)[2] | | | |
|---|---|---|---|---|---|
| | | Red Wine | White Wine | Rosé Wine | Sparkling Wine |
| TFA[3] | 10 - 2500 | 3.33 | 4.00 | 5.77 | 3.42 |
| PFPrA | 1 - 1000 | 1.67 | 1.46 | 0.87 | 1.18 |
| PFBA | 1 - 1000 | 1.00 | 1.11 | 1.88 | 0.92 |
| PFPeA | 1 - 1000 | 2.00 | 1.52 | 1.85 | 1.43 |
| PFHxA | 1 - 1000 | 2.10 | 1.54 | 1.95 | 1.87 |
| PFHpA | 1 - 1000 | 1.50 | 1.38 | 1.62 | 1.34 |
| PFOA | 1 - 1000 | 2.02 | 2.12 | 1.98 | 1.85 |
| PFNA | 1 - 1000 | 1.33 | 0.86 | 0.72 | 0.79 |
| PFDA | 1 - 1000 | 1.30 | 0.91 | 1.00 | 0.92 |
| PFUnA | 1 - 1000 | 1.04 | 0.92 | 0.88 | 0.92 |
| PFDoA | 1 - 1000 | 0.95 | 0.75 | 0.99 | 1.00 |
| PFTrDA | 1 - 1000 | 1.82 | 1.50 | 1.50 | 1.55 |
| TFMS | 1 - 1000 | 0.47 | 0.67 | 0.73 | 0.65 |

| | | | | | |
|---|---|---|---|---|---|
| PFEtS | 1 - 1000 | 0.70 | 0.67 | 0.86 | 0.75 |
| PFPrS | 1 - 1000 | 1.01 | 1.22 | 1.97 | 1.23 |
| PFBS | 1 - 1000 | 1.11 | 2.05 | 1.95 | 1.52 |
| PFPeS | 1 - 1000 | 0.78 | 0.85 | 0.86 | 0.64 |
| PFHxS | 1 - 1000 | 2.00 | 2.10 | 2.01 | 1.97 |
| PFHpS | 1 - 1000 | 0.80 | 1.00 | 1.11 | 0.86 |
| PFOS | 1 - 1000 | 1.50 | 1.28 | 1.50 | 1.19 |
| PFNS | 1 - 1000 | 0.68 | 0.78 | 0.82 | 0.75 |
| PFDS | 1 - 1000 | 0.67 | 0.98 | 0.83 | 0.66 |
| PFUdS | 1 - 1000 | 0.44 | 0.50 | 0.45 | 0.56 |
| PFDoS | 1 - 1000 | 0.68 | 0.75 | 0.78 | 0.63 |
| PFTrDS | 1 - 1000 | 0.75 | 0.98 | 0.78 | 0.65 |
| 4:2 FTS | 1 - 1000 | 0.87 | 0.79 | 0.78 | 0.79 |
| 6:2 FTS | 1 - 1000 | 0.83 | 0.70 | 0.81 | 0.81 |
| 8:2 FTS | 1 - 1000 | 0.83 | 0.68 | 0.88 | 0.98 |
| 5:3 FTCA | 4 - 1000 | 10.20 | 12.30 | 11.90 | 10.90 |
| 7:3 FTCA | 2 - 1000 | 9.02 | 5.28 | 4.62 | 4.30 |
| FOSA | 1 - 1000 | 1.88 | 1.50 | 1.33 | 0.60 |
| NMeFOSA | 2 - 1000 | 4.23 | 3.00 | 3.00 | 4.01 |
| NEtFOSA | 2 - 1000 | 3.53 | 5.29 | 4.25 | 4.70 |
| NMeFOSAA | 4 - 1000 | 10.50 | 12.00 | 11.80 | 10.25 |
| NEtFOSAA | 4 - 1000 | 8.55 | 11.50 | 10.20 | 10.21 |
| PFMPA | 1 - 1000 | 3.33 | 2.14 | 2.40 | 2.01 |
| PFMBA | 1 - 1000 | 2.00 | 1.32 | 1.11 | 1.21 |
| HFPO-DA | 10 - 1000 | 16.30 | 15.70 | 13.04 | 14.50 |
| ADONA | 1 - 1000 | 1.11 | 1.20 | 1.00 | 1.05 |
| PFEESA | 1 - 1000 | 0.44 | 0.41 | 0.44 | 0.45 |
| 9Cl-PF3ONS | 1 - 1000 | 0.41 | 0.55 | 0.41 | 0.52 |
| 11Cl-PF3OUdS | 1 - 1000 | 0.30 | 0.27 | 0.20 | 0.23 |

[1] Calibration range of standard solutions

[2] Concentration in wine samples

[3] LOD of TFA was determined with mass-labeled $^{13}C_2$-TFA

**Table S3.** Accuracy and precision for the analysis of fortified wine samples

## Red Wine:

| Analytes | Average Recovery (RSD, %), n=9 | | | | | |
|---|---|---|---|---|---|---|
| | Red Wine | | | | | |
| | Fortified Concentration (ng/L) | | | | | |
| | 2 | 4 | 10 | 20 | 100 | 500 |
| $^{13}C_2$-TFA | - | - | - | 89.5 (7.06) | 92.0 (8.36) | 103 (9.50) |
| PFPrA | - | - | 108 (9.10) | 105 (7.64) | 102 (3.19) | 105 (2.57) |
| PFBA | - | - | 100 (6.72) | 98.1 (6.63) | 97.8 (2.26) | 102 (3.22) |
| PFPeA | - | 106 (9.35) | 104 (7.38) | 104 (8.15) | 103 (8.26) | 105 (8.11) |
| PFHxA | - | 101 (6.24) | 104 (10.3) | 102 (9.37) | 100 (8.43) | 104 (9.73) |
| PFHpA | - | 107 (7.27) | 103 (9.82) | 100 (9.64) | 90.8 (6.87) | 96.1 (12.7) |
| PFOA | - | 98.6 (7.81) | 94.5 (7.60) | 97.6 (7.09) | 89.6 (9.33) | 89.6 (9.43) |
| PFNA | - | 97.9 (5.89) | 102 (9.39) | 102 (8.46) | 104 (5.96) | 106 (7.86) |
| PFDA | - | 110 (3.71) | 101 (6.87) | 103 (5.45) | 99.6 (12.7) | 104 (12.7) |
| PFUnA | 99.8 (7.56) | 103 (8.99) | 102 (11.5) | 102 (14.5) | 107 (11.8) | 107 (15.2) |
| PFDoA | 106 (11.0) | 102 (10.4) | 109 (6.06) | 103 (11.5) | 108 (12.1) | 108 (9.86) |
| PFTrDA | - | 104 (2.27) | 107 (5.03) | 103 (6.93) | 107 (10.8) | 110 (6.81) |
| TFMS | - | - | 112 (6.01) | 103 (4.12) | 103 (5.90) | 110 (4.73) |
| PFEtS | 98.0 (2.72) | 119 (6.22) | 132 (6.81) | 128 (4.26) | 125 (5.41) | 129 (6.01) |
| PFPrS | 104 (10.0) | 94.8 (9.24) | 101 (10.6) | 93.3 (8.48) | 92.1 (7.63) | 90.9 (7.41) |
| PFBS | 111 (8.01) | 107 (6.51) | 108 (5.74) | 99.7 (9.20) | 106 (4.47) | 107 (5.17) |
| PFPeS | 98.4 (7.95) | 97.7 (9.11) | 102 (10.3) | 95.5 (15.0) | 94.5 (9.37) | 86.2 (5.50) |
| PFHxS | - | 104 (8.84) | 95.5 (6.83) | 99.1 (8.61) | 100 (4.95) | 99.8 (6.20) |
| PFHpS | 101 (6.26) | 97.9 (5.69) | 106 (8.75) | 107 (11.9) | 106 (8.57) | 112 (7.55) |
| PFOS | - | 95.6 (5.75) | 93.2 (6.90) | 96.9 (6.94) | 108 (7.25) | 110 (5.51) |
| PFNS | 100 (11.9) | 102 (9.32) | 106 (6.15) | 103 (9.04) | 101 (10.2) | 109 (6.52) |
| PFDS | 111 (10.8) | 102 (7.43) | 101 (10.2) | 97.5 (7.88) | 98.7 (6.70) | 101 (7.27) |
| PFUdS | 101 (11.0) | 96.3 (11.9) | 99.4 (12.8) | 92.3 (6.28) | 93.4 (10.4) | 97.8 (11.7) |
| PFDoS | 100 (9.43) | 98.7 (5.93) | 107 (9.47) | 95.9 (8.58) | 94.6 (10.9) | 102 (8.91) |
| PFTrDS | 104 (10.3) | 98.5 (10.8) | 100 (8.23) | 95.7 (11.8) | 95.6 (8.49) | 101 (9.01) |
| 4:2 FTS | 93.2 (9.48) | 98.6 (11.4) | 100 (11.1) | 97.6 (11.3) | 101 (12.2) | 99.4 (13.2) |
| 6:2 FTS | 101 (9.07) | 104 (8.58) | 110 (3.25) | 103 (10.2) | 102 (9.28) | 100 96.85) |
| 8:2 FTS | 89.6 (9.57) | 87.2 (6.62) | 90.2 (6.23) | 92.2 (5.22) | 90.9 (5.62) | 91.9 (6.62) |
| FOSA | - | 90.1 (10.5) | 103 (9.14) | 99.8 (11.0) | 108 (9.81) | 107 (9.96) |

| | | | | | | |
|---|---|---|---|---|---|---|
| NMeFOSA | - | - | 103 (8.40) | 101 (10.2) | 95.6 (12.6) | 87.4 (7.64) |
| NEtFOSA | - | - | 99.2 (9.54) | 92.2 (10.7) | 87.9 (10.0) | 90.3 (5.85) |
| 5:3 FTCA | - | - | - | 106 (7.56) | 98.8 (11.6) | 88.3 (6.61) |
| 7:3 FTCA | - | - | - | 98.0 (6.60) | 85.2 (4.81) | 84.9 (4.58) |
| NMeFOSAA | - | - | - | 90.7 (6.73) | 92.5 (9.81) | 101 (12.8) |
| NEtFOSAA | - | - | 109 (5.85) | 93.3 (13.6) | 97.0 (13.5) | 100 (14.2) |
| PFMPA | - | 104 (7.14) | 100 (8.32) | 102 (8.35) | 102 (4.60) | 110 (2.57) |
| PFMBA | - | 109 (7.64) | 101 (7.53) | 99.5 (8.19) | 109 (6.55) | 113 (3.24) |
| HFPO-DA | - | - | - | 109 (5.04) | 106 (7.36) | 99.8 (7.06) |
| ADONA | - | 98.1 (5.12) | 98.9 (8.91) | 98.9 (8.65) | 104 (9.99) | 106 (8.09) |
| PFEESA | 98.5 (9.11) | 94.3 (9.17) | 102 (7.16) | 107 (6.38) | 111 (6.39) | 105 (3.94) |
| 9Cl-PF3ONS | 100 (10.9) | 102 (7.47) | 89.1 (6.77) | 91.7 (9.03) | 103 (11.3) | 97.5 (6.49) |
| 11Cl-PF3OUdS | 107 (8.93) | 109 (7.53) | 108 (9.05) | 113 (2.99) | 107 (8.55) | 110 (7.99) |

## White Wine:

| Analytes | Average Recovery (RSD, %), n=9 | | | | | |
|---|---|---|---|---|---|---|
| | White Wine | | | | | |
| | Fortified Concentration (ng/L) | | | | | |
| | 2 | 4 | 10 | 20 | 100 | 500 |
| $^{13}C_2$-TFA | - | - | - | 81.7 (7.12) | 85.7 (5.91) | 95.8 (9.29) |
| PFPrA | - | - | 100 (11.9) | 96.4 (4.82) | 102 (1.73) | 101 (3.89) |
| PFBA | - | - | 99.7 (10.3) | 91.8 (7.85) | 101 (5.97) | 100 (2.87) |
| PFPeA | - | 107 (7.75) | 92.6 (6.34) | 95.7 (7.63) | 99.4 (4.44) | 105 (3.55) |
| PFHxA | - | 101 (7.17) | 95.8 (7.18) | 99.7 (8.09) | 94.6 (8.95) | 97.3 (4.38) |
| PFHpA | - | 92.4 (12.5) | 99.3 (10.9) | 92.6 (8.16) | 105 (4.49) | 106 (5.04) |
| PFOA | - | 96.2 (12.6) | 93.9 (4.72) | 84.5 (4.59) | 87.0 (8.71) | 86.2 (3.97) |
| PFNA | 98.5 (9.24) | 99.2 (8.79) | 97.5 (10.7) | 95.5 (6.52) | 97.6 (9.50) | 98.7 (8.99) |
| PFDA | 105 (11.1) | 111 (3.38) | 113 (5.94) | 108 (8.09) | 114 (3.53) | 116 (3.20) |
| PFUnA | 99.0 (7.75) | 102 (9.25) | 102 (8.47) | 96.9 (9.31) | 104 (8.92) | 101 (11.6) |
| PFDoA | 100 (12.1) | 98.6 (11.1) | 104 (10.0) | 100 (13.2) | 109 (5.97) | 104 (5.38) |
| PFTrDA | - | 96.4 (8.76) | 100 (9.04) | 101 (9.13) | 110 (7.01) | 109 (4.52) |
| TFMS | - | - | 107 (4.30) | 96.9 (5.02) | 102 (4.69) | 103 (6.54) |
| PFEtS | 148 (11.4) | 142 (9.94) | 144 (9.20) | 138 (4.57) | 140 (3.32) | 145 (3.67) |
| PFPrS | 106 (12.8) | 100 (6.62) | 89.6 (6.44) | 84.2 (5.11) | 84.9 (3.89) | 82.5 (2.85) |
| PFBS | - | 108 (7.49) | 109 (10.4) | 104 (5.39) | 106 (5.33) | 105 (5.38) |
| PFPeS | 107 (6.78) | 92.9 (7.80) | 96.3 (7.44) | 86.2 (4.97) | 96.3 (4.78) | 85.7 (7.94) |

| | | | | | | |
|---|---|---|---|---|---|---|
| PFHxS | - | 109 (9.92) | 96.3 (11.9) | 93.9 (6.36) | 98.3 (5.66) | 102 (7.26) |
| PFHpS | 100 (13.0) | 101 (6.37) | 106 (9.14) | 101 (6.21) | 108 (7.25) | 113 (3.61) |
| PFOS | - | 110 (6.35) | 95.4 (6.96) | 102 (6.12) | 100 (5.45) | 107 (4.71) |
| PFNS | 100 (8.49) | 98.1 (7.30) | 98.7 (10.0) | 96.0 (12.0) | 98.9 (10.9) | 102 (9.27) |
| PFDS | 99.5 (10.9) | 96.0 (6.56) | 110 (7.07) | 103 (8.76) | 110 (4.03) | 107 (6.86) |
| PFUdS | 101 (7.69) | 95.6 (7.49) | 105 (10.8) | 97.9 (12.9) | 104 (11.4) | 99.4 (13.0) |
| PFDoS | 99.5 (8.09) | 104 (9.81) | 101 (9.48) | 104 (11.8) | 109 (9.05) | 104 (12.9) |
| PFTrDS | 99.8 (11.4) | 94.3 (8.73) | 96.7 (8.72) | 92.1 (7.66) | 99.9 (5.66) | 94.3 (4.67) |
| 4:2 FTS | 93.8 (10.0) | 89.6 (11.5) | 88.6 (12.4) | 87.7 (6.37) | 96.1 (9.98) | 85.1 (4.85) |
| 6:2 FTS | 99.6 (11.5) | 97.8 (11.9) | 103 (6.25) | 104 (4.02) | 108 (4.86) | 101 (6.46) |
| 8:2 FTS | 109 (9.21) | 99.7 (9.57) | 106 (3.42) | 96.0 (4.83) | 103 (3.90) | 97.3 (2.40) |
| FOSA | - | 94.7 (12.3) | 106 (9.40) | 95.7 (9.01) | 109 (6.25) | 114 (5.41) |
| NMeFOSA | - | - | 109 (7.93) | 102 (6.83) | 110 (4.86) | 111 (7.02) |
| NEtFOSA | - | - | 107 (8.47) | 95.5 (12.3) | 103 (7.83) | 102 (9.24) |
| 5:3 FTCA | - | - | - | 95.0 (8.63) | 91.1 (12.8) | 87.7 (8.41) |
| 7:3 FTCA | - | - | 90.7 (5.43) | 92.1 (7.89) | 88.7 (9.66) | 90.4 (7.36) |
| NMeFOSAA | - | - | - | 112 (10.1) | 99.2 (9.17) | 112 (5.89) |
| NEtFOSAA | - | - | - | 98.2 (12.7) | 96.9 (11.5) | 108 (5.97) |
| PFMPA | - | 98.4 (6.65) | 100 (10.4) | 93.5 (8.74) | 105 (4.92) | 105 (4.41) |
| PFMBA | 108 (9.64) | 107 (6.91) | 99.0 (9.54) | 98.5 (9.40) | 102 (8.54) | 102 (9.22) |
| HFPO-DA | - | - | - | 99.7 (8.33) | 106 (10.4) | 114 (6.17) |
| ADONA | 99.2 (12.4) | 99.2 (11.1) | 98.9 (7.37) | 99.3 (11.7) | 99.6 (12.8) | 97.3 (13.7) |
| PFEESA | 109 (9.34) | 108 (8.35) | 105 (7.09) | 97.8 (5.95) | 102 (3.43) | 103 (6.55) |
| 9Cl-PF3ONS | 102 (9.25) | 91.2 (11.5) | 94.0 (7.93) | 84.2 (5.03) | 86.1 (3.06) | 86.7 (2.40) |
| 11Cl-PF3OUdS | 116 (4.64) | 111 (3.94) | 116 (3.35) | 103 (1.83) | 110 (1.70) | 110 (1.51) |

## Rosé Wine:

| Analytes | Average Recovery (RSD, %), n=9 | | | | | |
|---|---|---|---|---|---|---|
| | Rosé Wine | | | | | |
| | Fortified Concentration (ng/L) | | | | | |
| | 2 | 4 | 10 | 20 | 100 | 500 |
| $^{13}C_2$-TFA | - | - | - | 89.4 (8.56) | 82.8 (4.48) | 90.9 (10.5) |
| PFPrA | - | - | 110 (7.75) | 105 (7.08) | 106 (3.09) | 105 (1.32) |
| PFBA | - | - | 102 (7.16) | 97.9 (5.62) | 104 (3.09) | 102 (4.28) |
| PFPeA | - | 106 (9.15) | 104 (5.98) | 89.3 (8.23) | 93.2 (6.05) | 98.7 (5.25) |

| | | | | | | |
|---|---|---|---|---|---|---|
| PFHxA | - | 101 (9.86) | 95.9 (6.58) | 94.8 (6.78) | 89.1 (3.84) | 95.6 (5.22) |
| PFHpA | - | 102 (6.57) | 100 (8.46) | 98.8 (9.71) | 92.4 (5.23) | 86.3 (4.84) |
| PFOA | - | 98.9 (8.01) | 98.0 (8.75) | 104 (9.41) | 97.0 (7.39) | 96.9 (7.20) |
| PFNA | 97.7 (6.77) | 100 (7.88) | 97.7 (6.54) | 97.0 (5.09) | 105 (9.78) | 110 (11.7) |
| PFDA | 104 (3.45) | 100 (3.14) | 95.2 (5.14) | 98.1 (7.41) | 99.0 (4.82) | 102 (5.92) |
| PFUnA | 94.2 (8.91) | 92.4 (9.24) | 89.4 (8.88) | 88.8 (6.70) | 93.3 (9.98) | 93.4 (12.0) |
| PFDoA | 107 (10.3) | 96.8 (6.62) | 102 (7.52) | 100 (7.73) | 111 (5.88) | 114 (7.29) |
| PFTrDA | - | 108 (8.87) | 99.9 (7.85) | 98.0 (4.20) | 102 (6.46) | 107 (7.64) |
| TFMS | - | - | 108 (6.68) | 102 (4.90) | 102 (4.05) | 108 (5.48) |
| PFEtS | 144 (4.68) | 142 (6.88) | 134 (8.21) | 133 (8.42) | 141 (9.29) | 138 (9.66) |
| PFPrS | - | 87.3 (4.42) | 92.8 (9.29) | 92.5 (6.12) | 91.3 (5.06) | 96.7 (5.24) |
| PFBS | - | 114 (3.48) | 102 (6.91) | 99.0 (7.27) | 96.3 (5.02) | 98.5 (6.18) |
| PFPeS | - | 88.0 (7.48) | 88.7 (6.45) | 87.7 (7.57) | 86.5 (5.45) | 84.3 (3.42) |
| PFHxS | - | 109 (12.1) | 102 (6.99) | 99.1 (7.59) | 95.1 (9.21) | 98.8 (6.09) |
| PFHpS | 89.4 (9.06) | 92.3 (11.6) | 95.5 (9.97) | 99.7 (10.1) | 96.6 (11.4) | 107 (9.64) |
| PFOS | - | 112 (5.87) | 92.9 (6.56) | 95.6 (7.75) | 97.4 (9.92) | 108 (5.56) |
| PFNS | 114 (9.64) | 102 (7.58) | 95.1 (7.78) | 93.7 (7.27) | 98.1 (6.78) | 108 (11.2) |
| PFDS | 110 (10.0) | 96.0 (9.74) | 96.1 (8.73) | 91.5 (7.64) | 101 (8.82) | 105 (6.83) |
| PFUdS | 92.6 (7.50) | 93.0 (7.66) | 95.0 (7.31) | 98.4 (5.44) | 107 (4.91) | 110 (6.51) |
| PFDoS | 100 (7.46) | 101 (10.2) | 100 (9.36) | 101 (7.41) | 110 (4.75) | 111 (4.64) |
| PFTrDS | 107 (9.22) | 105 (7.03) | 96.0 (5.72) | 97.0 (8.72) | 112 (3.87) | 114 (3.70) |
| 4:2 FTS | 88.1 (9.75) | 86.1 (7.30) | 82.5 (6.78) | 86.0 (6.55) | 87.5 (6.02) | 84.2 (5.20) |
| 6:2 FTS | 88.9 (9.92) | 95.8 (9.76) | 87.2 (9.12) | 94.0 (7.81) | 102 (6.21) | 93.3 (5.82) |
| 8:2 FTS | 94.2 (9.25) | 86.0 (7.62) | 85.4 (7.02) | 86.4 (6.63) | 93.7 (6.31) | 93.2 (6.60) |
| FOSA | - | 92.1 (9.27) | 103 (10.5) | 98.2 (7.64) | 104 (6.64) | 111 (4.49) |
| NMeFOSA | - | - | 110 (8.45) | 101 (6.72) | 103 (8.29) | 102 (8.44) |
| NEtFOSA | - | - | 107 (7.58) | 93.9 (11.6) | 97.1 (8.87) | 107 (7.21) |
| 5:3 FTCA | - | - | - | 109 (7.32) | 105 (11.0) | 105 (9.88) |
| 7:3 FTCA | - | - | 98.9 (8.59) | 102 (7.94) | 95.9 (3.67) | 91.9 (4.51) |
| NMeFOSAA | - | - | - | 84.7 (6.70) | 87.4 (5.28) | 98.7 (7.03) |
| NEtFOSAA | - | - | - | 98.5 (9.43) | 91.2 (10.2) | 92.1 (9.41) |
| PFMPA | - | 104 (8.72) | 98.8 (8.38) | 93.6 (7.04) | 96.3 (3.68) | 103 (6.11) |
| PFMBA | 100 (9.35) | 98.6 (6.51) | 94.1 (8.62) | 92.5 (10.6) | 90.3 (10.3) | 102 (6.40) |
| HFPO-DA | - | - | - | 104 (8.26) | 99.1 (8.68) | 100 (11.6) |
| ADONA | - | 95.8 (6.67) | 90.7 (5.82) | 85.5 (6.78) | 85.7 (4.30) | 86.2 (6.98) |
| PFEESA | 98.4 (7.51) | 99.2 (8.21) | 94.2 (8.50) | 93.3 (7.64) | 97.0 (4.47) | 102 (6.74) |

| | | | | | | |
|---|---|---|---|---|---|---|
| 9Cl-PF3ONS | 102 (11.8) | 90.8 (6.58) | 89.1 (11.1) | 82.1 (5.12) | 87.0 (7.40) | 92.7 (9.15) |
| 11Cl-PF3OUdS | 99.2 (9.08) | 94.8 (10.9) | 93.2 (10.0) | 94.6 (9.12) | 101 (10.8) | 105 (11.5) |

## Sparkling Wine:

| Analytes | Average Recovery (RSD, %), n=9 | | | | | |
|---|---|---|---|---|---|---|
| | Sparkling Wine | | | | | |
| | Fortified Concentration (ng/L) | | | | | |
| | 2 | 4 | 10 | 20 | 100 | 500 |
| $^{13}C_2$-TFA | - | - | - | 95.9 (12.8) | 90.7 (11.3) | 104 (12.7) |
| PFPrA | - | - | 99.1 (10.2) | 97.9 (7.25) | 103 (4.05) | 103 (3.87) |
| PFBA | - | - | 101 (9.87) | 100 (8.63) | 102 (5.45) | 103 (4.83) |
| PFPeA | - | 99.6 (6.96) | 99.1 (8.01) | 91.8 (8.59) | 86.8 (4.66) | 91.1 (5.82) |
| PFHxA | - | 100 (8.86) | 95.9 (7.12) | 100 (11.6) | 97.4 (5.97) | 101 (4.03) |
| PFHpA | 104 (11.2) | 104 (7.41) | 105 (6.90) | 97.7 (8.56) | 97.9 (6.82) | 101 (7.61) |
| PFOA | - | 94.0 (9.79) | 106 (5.89) | 102 (7.82) | 97.7 (8.84) | 94.6 (5.62) |
| PFNA | 105 (8.27) | 98.3 (9.13) | 105 (7.93) | 104 (3.97) | 104 (5.03) | 106 (3.02) |
| PFDA | 107 (8.10) | 103 (10.0) | 99.5 (11.2) | 99.5 (12.7) | 110 (6.90) | 109 (5.71) |
| PFUnA | 107 (10.2) | 112 (6.87) | 112 (5.97) | 106 (7.76) | 111 (7.00) | 114 (3.48) |
| PFDoA | 103 (6.16) | 102 (8.35) | 111 (5.38) | 111 (2.01) | 119 (2.17) | 119 (3.23) |
| PFTrDA | - | 99.7 (13.0) | 102 (7.69) | 101 (5.09) | 110 (6.55) | 113 (3.49) |
| TFMS | - | - | 91.3 (6.08) | 98.6 (8.27) | 98.2 (9.26) | 103 (7.91) |
| PFEtS | 141 (6.25) | 137 (7.64) | 151 (3.98) | 145 (4.77) | 152 (3.91) | 154 (4.42) |
| PFPrS | - | 91.7 (7.37) | 102 (6.43) | 98.3 (6.96) | 94.5 (6.41) | 93.5 (12.1) |
| PFBS | - | 105 (6.71) | 100 (7.27) | 99.5 (5.36) | 99.5 (4.67) | 99.0 (5.07) |
| PFPeS | 98.4 (11.9) | 94.3 (6.91) | 93.2 (12.7) | 87.0 (5.48) | 86.7 (7.49) | 90.3 (7.83) |
| PFHxS | - | 102 (9.46) | 106 (10.8) | 93.3 (7.78) | 92.0 (7.33) | 93.7 (9.62) |
| PFHpS | 101 (10.9) | 97.7 (8.26) | 99.0 (8.71) | 101 (13.1) | 106 (8.33) | 110 (5.48) |
| PFOS | - | 97.6 (9.12) | 97.6 (9.53) | 101 (10.5) | 105 (7.82) | 108 (6.61) |
| PFNS | 92.9 (9.43) | 91.4 (6.73) | 95.0 (9.16) | 89.0 (7.51) | 91.3 (11.7) | 92.0 (9.53) |
| PFDS | 113 (8.33) | 104 (10.7) | 107 (9.88) | 104 (10.5) | 109 (10.4) | 112 (5.13) |
| PFUdS | 116 (6.45) | 110 (5.11) | 108 (6.32) | 95.8 (7.24) | 97.6 (7.94) | 105 (5.70) |
| PFDoS | 99.3 (13.4) | 99.0 (13.7) | 109 (6.61) | 94.8 (6.41) | 109 (6.67) | 114 (2.53) |
| PFTrDS | 108 (13.4) | 104 (13.5) | 112 (6.50) | 97.0 (12.2) | 110 (6.43) | 116 (2.99) |
| 4:2 FTS | - | 90.8 (11.8) | 89.8 (11.7) | 92.8 (8.44) | 108 (8.15) | 99.2 (7.47) |
| 6:2 FTS | 87.4 (7.12) | 95.2 (7.23) | 95.2 (10.8) | 87.6 (4.69) | 102 (5.49) | 101 (7.20) |

| | | | | | | |
|---|---|---|---|---|---|---|
| 8:2 FTS | 98.8 (10.0) | 102 (7.99) | 104 (9.20) | 102 (9.01) | 115 (3.01) | 114 (4.88) |
| FOSA | 107 (10.0) | 100 (13.3) | 103 (13.6) | 108 (10.3) | 112 (6.33) | 116 (2.54) |
| NMeFOSA | - | - | 104 (9.75) | 102 (8.55) | 114 (7.18) | 116 (3.81) |
| NEtFOSA | - | - | 110 (8.75) | 94.9 (9.99) | 109 (7.01) | 117 (4.59) |
| 5:3 FTCA | - | - | - | 108 (6.94) | 110 (7.74) | 111 (5.63) |
| 7:3 FTCA | - | - | 103 (7.55) | 98.5 (7.00) | 99.9 (9.04) | 99.0 (10.0) |
| NMeFOSAA | - | - | - | 103 (12.9) | 97.7 (11.8) | 104 (5.91) |
| NEtFOSAA | - | - | - | 95.7 (12.8) | 96.8 (10.2) | 114 (4.35) |
| PFMPA | - | 96.0 (8.68) | 93.4 (5.25) | 93.9 (10.8) | 97.0 (4.85) | 100 (6.09) |
| PFMBA | 97.4 (10.0) | 94.5 (10.3) | 99.0 (7.68) | 95.6 (5.64) | 107 (6.68) | 112 (4.89) |
| HFPO-DA | - | - | - | 109 (10.2) | 91.6 (11.8) | 89.8 (9.98) |
| ADONA | - | 95.4 (9.07) | 97.5 (11.1) | 92.2 (7.04) | 92.5 (11.9) | 101 (12.7) |
| PFEESA | 104 (11.2) | 107 (9.31) | 108 (5.90) | 103 (7.51) | 101 (5.68) | 102 (5.95) |
| 9Cl-PF3ONS | 90.3 (12.5) | 91.0 (11.3) | 95.6 (8.68) | 89.3 (8.82) | 95.1 (7.25) | 96.6 (5.84) |
| 11Cl-PF3OUdS | 101 (12.4) | 104 (11.1) | 94.1 (11.0) | 95.6 (10.2) | 97.4 (8.51) | 97.5 (7.82) |

**Table S4**. Measurement of PFAS in commercial wine samples

| Wine Samples | Vintage Year | Closure Type | Concentration (ng/L) | | | |
|---|---|---|---|---|---|---|
| | | | TFA | PFPrA | PFBA | TFMS |
| ***Red Wine*** | | | | | | |
| Red #1 (USA) | Non-Vintage | Screw Cap | 21,998 | 65.5 | 24.4 | 10.0 |
| Red #2 (USA) | 2023 | Natural Cork | 109,503 | 59.3 | 100.2 | 27.1 |
| Red #3 (USA) | Non-Vintage | Natural Cork | 99,320 | 220.1 | 163.6 | 24.8 |
| Red #4 (USA) | Non-Vintage | Screw Cap | 153,673 | 57.9 | 97.0 | 19.0 |
| Red #5 (USA) | 2024 | Synthetic Cork | 28,697 | 290.0 | 81.0 | 38.7 |
| Red #6 (USA) | 2022 | Natural Cork | 103,707 | 225.1 | 101.4 | 20.5 |
| Red #7 (USA) | Non-Vintage | Screw Cap | 104,591 | 177.5 | 92.7 | 13.8 |
| Red #8 (USA) | Non-Vintage | Screw Cap | 40,526 | 88.8 | 51.3 | 11.6 |
| Red #9 (Australia) | 2022 | Screw Cap | 53,908 | 34.2 | 89.0 | 7.4 |
| Red #10 (France) | 2022 | Natural Cork | 38,143 | 69.1 | 45.9 | 7.7 |
| ***White Wine*** | | | | | | |
| White #1 (USA) | Non-Vintage | Screw Cap | 124,322 | 333.6 | 153.6 | 20.0 |
| White #2 (USA) | Non-Vintage | Screw Cap | 68,349 | 231.7 | 126.2 | 22.9 |
| White #3 (France) | 2022 | Natural Cork | 168,666 | 59.1 | 44.8 | 9.8 |
| White #4 (Italy) | 2024 | Screw Cap | 24,280 | 89.6 | 29.6 | 6.2 |
| White #5 (New Zealand) | 2025 | Screw Cap | 406,350 | 29.1 | 45.7 | 6.4 |
| ***Rosé Wine*** | | | | | | |
| Rosé #1 (USA) | Non-Vintage | Screw Cap | 93,514 | 122.0 | 65.9 | 13.0 |
| Rosé #2 (USA) | Non-Vintage | Screw Cap | 96,993 | 144.5 | 69.4 | 28.7 |
| Rosé #3 (USA) | 2023 | Screw Cap | 67,880 | 151.6 | 82.9 | 20.6 |
| Rosé #4 (France) | 2025 | Glass Stopper | 100,224 | 319.1 | 62.0 | 47.2 |
| Rosé #5 (Italy) | Non-Vintage | Screw Cap | 20,777 | 107.3 | 14.7 | 22.4 |
| ***Sparkling Wine*** | | | | | | |
| Sparkling #1 (USA) | Non-Vintage | Natural Cork | 45,649 | 104.3 | 61.4 | 9.5 |
| Sparkling #2 (Italy) | Non-Vintage | Agglomerated Cork | 56,801 | 64.7 | 26.8 | 10.2 |
| Sparkling #3 (Italy) | Non-Vintage | Agglomerated Cork | 27,010 | 83.2 | 43.6 | 69.6 |
| Sparkling #4 (Italy) | Non-Vintage | Agglomerated Cork | 25,667 | 48.9 | 13.8 | 35.0 |
| Sparkling #4 (Italy) | Non-Vintage | Agglomerated Cork | 29,131 | 495.2 | 400.5 | 25.4 |

**Table S5.** Alternative LC method for confirmatory analysis of TFA, PFPrA, and PFBA in wine

| | | |
|---|---|---|
| **Column** | Ultra IBD Inert 100 × 2.1 mm, 3.0 µm | |
| **Delay Column** | Ultra IBD 150 × 2.1 mm, 3.0 µm | |
| **Mobile Phase A** | 0.75 mM ammonium formate, 0.1% formic acid in water | |
| **Mobile Phase B** | 0.75 mM ammonium formate, 0.1% formic acid in 95:5 acetonitrile:water | |
| **Gradient** | **Time (min)** | **%B** |
| | 0.00 | 50 |
| | 9.00 | 100 |
| | 10.00 | 100 |
| | 10.01 | 50 |
| | 12.00 | 50 |
| **Flow Rate** | 0.4 mL/min | |
| **Injection Volume** | 20 µL | |
| **Column Temp.** | 40 °C | |

**Figure S1.** Confirmatory chromatographic comparison of (A) original and (B) alternative LC methods for TFA, PFPrA, and PFBA in a red wine sample

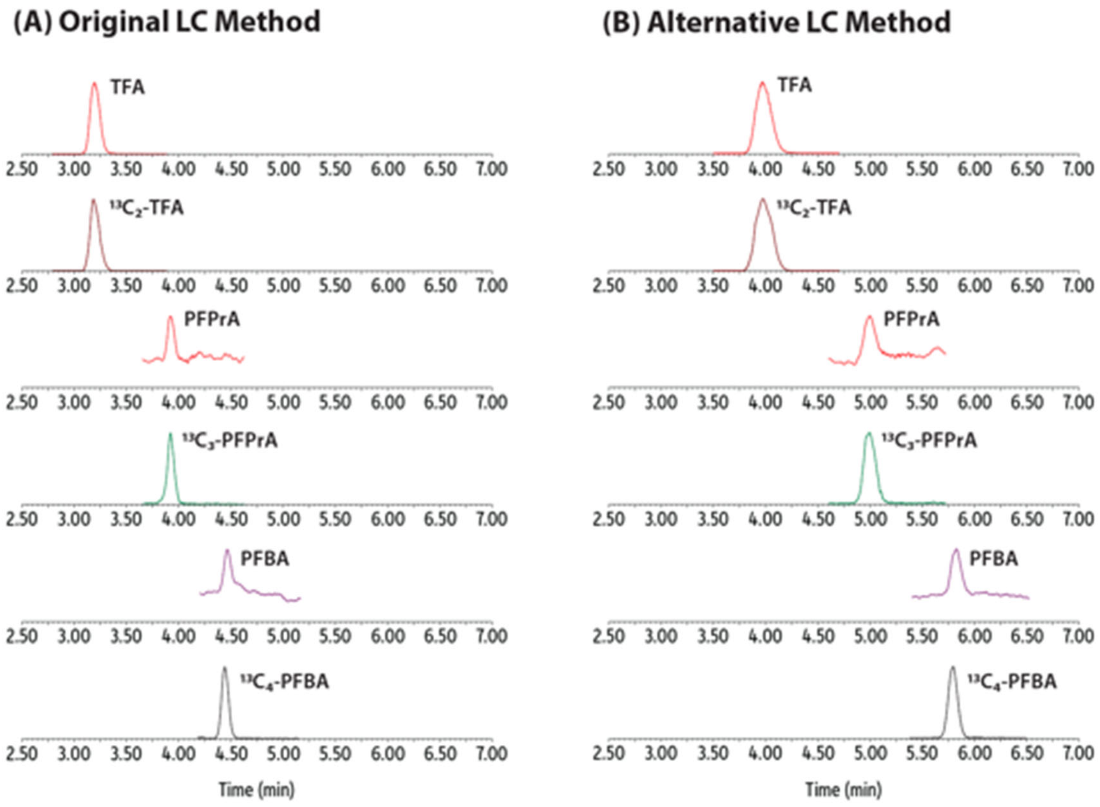

Supplement: Supplementary file 1 [file jox-16-00171-s001.zip › jox-4473421-supplementary.pdf]
